# The Alice Memorial Hospital, Hong Kong

**Published:** 1892-04-09

**Authors:** 


### THE ALICE MEMORIAL HOSPITAL, HONG KONG.

A Hint to the London Missionary Society.

Dr. John C. Thomson in his report of the Alice Memorial Hospital, Hong Kong, which contains 90 beds, and treats 848 in-patients, and 8,929 out-patients yearly, calls attention to the necessity of removing the hospital from its present site on hygienic grounds. It is shut in on the north and east by Chinese houses, and on the south and west by narrow streets. Formerly the site was fairly open on the north towards the harbour, but this has been closed by the erection of a pile of Chinese houses. It is stated, though the reason does not appear, that "a great deal of washing of soiled dressings has to be done on the spot in a closed courtyard, from which gas and vapours have no means of escape except skywards, after passing the open doors and windows of all the wards." No wonder that "the health of the hospital is not such as it ought to be." Indeed, seeing that the institution is in connection with the London Missionary Society, the society ought either to supply the necessary funds to enable the work of the hospital to be done efficiently and without danger to the lives of the patients, or to order it to be closed. They cannot for their own credit's sake allow their name to be associated much longer with so insanitary an institution of which Dr. Thomson reports as follows: "In spite of the continuous abundant use of antiseptics and disenfectants, foul-smelling odours arise from this courtyard, and are bound to injure the health of the inmates," and again "fevers of a malarial type often arise within the walls. . . . while many operation cases have their convalescence interrupted by inter-current attacks of fever that in more favourable circumstances would probably not occur." Having regard to the reserve under which Dr. Thomson necessarily writes it is not difficult to realise the grave dangers of the present situation in this hospital, and we would impress upon the authorities of the London Missionary Society the urgent importance which exists for immediate action in the matter.

